# Nanoengineering Systems for Gene Therapy: Mechanisms, Modalities, and Future Directions

**DOI:** 10.3390/ijms27135988

**Published:** 2026-07-03

**Authors:** Raheem Mais, Ayush Kumar, Armand Ahmetaj, Gaby Burgos-Crespo, Mary Margarette Sanchez, Dianne Claire Roxas, Christopher Dcosta, Azhar Ilyas, Michael Hadjiargyrou, Steven Zanganeh

**Affiliations:** 1 Department of Bioengineering, New York Institute of Technology, New York, NY 11568, USA; amais@nyit.edu (R.M.); akumar42@nyit.edu (A.K.); aahmet03@nyit.edu (A.A.); gburgosc@nyit.edu (G.B.-C.); msanch11@nyit.edu (M.M.S.); droxas@nyit.edu (D.C.R.); cdcosta@nyit.edu (C.D.); ailyas@nyit.edu (A.I.); 2 Department of Biological & Chemical Sciences, New York Institute of Technology, New York, NY 11568, USA; mhadji@nyit.edu

**Keywords:** nanotechnology, gene therapy, genome engineering, CRISPR-Cas systems, nanomaterials, targeted delivery, genetic disorders

## Abstract

Nanotechnology has become an important platform in the fields of gene therapy and genome editing, providing delivery strategies that address persistent therapeutic challenges by improving the precision, efficiency, and safety of genetic modifications. This review highlights the central role of nanomaterials in overcoming persistent barriers to genetic interventions, including inefficient delivery, instability of genetic cargo, and off-target effects. Specifically, we emphasize the combined use of nanomaterials with clustered regularly interspaced short palindromic repeats and CRISPR-associated proteins (CRISPR-Cas) systems, which can improve editing specificity and therapeutic efficacy. Beyond the classical CRISPR/Cas9 platform, this review also discusses next-generation modalities such as base editors, Cas13, prime editing, and the recently described Tandem Interspaced Guide RNA and TIGR-associated protein (TIGR-Tas) system, while considering their therapeutic potential and distinct delivery challenges. By using nanomaterials, the stability and intracellular delivery of genome-editing systems are improved, enabling more effective treatments for genetic disorders and acquired diseases such as cancer and infectious diseases. In addition, nanocarriers provide controlled release, protection from degradation, and better biocompatibility, thereby improving the safety and reliability of gene-editing therapies. Despite these advances, important translational challenges remain, including immunotoxicity, large-scale manufacturing, and regulatory integration. Overall, the continued convergence of nanotechnology and genome engineering may support the development of personalized medicine strategies that adapt genetic engineering tools for patient-specific applications.

## 1. Introduction

Gene therapy has been recognized as a clinical option in modern medicine, with applications in treating monogenic diseases and B-cell malignancies [1]. However, traditional approaches still face critical limitations, including inefficient delivery, immune reactions, and risks of unintended genetic alterations [2]. The replacement of defective gene sequences hold therapeutic promise, but achieving targeted, safe, and efficient delivery of DNA to specific cells remains a significant challenge [3]. Recent investigations have shown that viral vectors are the most commonly used delivery systems, yet they are associated with immune activation and safety risks [4]. Moreover, viral integration can cause insertional mutagenesis, where therapeutic genes disrupt host genomic sequences, potentially leading to oncogenesis such as leukemia [5].

Genome engineering provides a powerful set of tools for precise addition, deletion, and correction of genes in situ and in vivo, extending applications from basic research to clinical medicine [6]. CRISPR and CRISPR-associated (Cas) proteins, in particular, have become transformative technologies for regulating cell function, enabling precise targeting, and altering the cellular microenvironment. Understanding these technologies has opened new avenues for precision therapies and improved diagnostics [7]. Over the past decade, CRISPR-based systems have rapidly expanded, with multiple studies demonstrating their ability to perform programmable editing in mammalian cells [8].

Among these, the most widely used platform is the type II CRISPR-Cas9 system from *Streptococcus pyogenes* (SpCas9), which introduces double-strand breaks (DSBs) at target sites [9]. Target recognition is mediated by a programmable guide RNA (gRNA), allowing highly specific cleavage adjacent to a protospacer adjacent motif (PAM, NGG sequence) [10]. Repair of Cas9-induced DSBs occurs through pathways such as homology-directed repair (HDR), non-homologous end joining (NHEJ), or microhomology-mediated end joining (MMEJ) [7]. HDR enables precise sequence replacement, while NHEJ and MMEJ frequently introduce insertions and deletions (indels), which can be exploited to disrupt coding or noncoding sequences [7].

A diverse range of Cas proteins from different bacterial species was developed to expand genome-editing capabilities [7] For example, *Staphylococcus aureus* Cas9 (SaCas9) recognizes different PAM sequences than SpCas9 and is small enough to be packaged into adeno-associated virus (AAV) vectors. Similarly, *Neisseria meningitidis* Cas9 (NmeCas9) and *Campylobacter jejuni* Cas9 (CjCas9) are also compact, facilitating viral delivery. In contrast, Cas12a introduces staggered DSBs and enables multiplex targeting, while Cas13 uniquely targets RNA rather than DNA [7,11].

Beyond these canonical systems, several next-generation genome editing platforms have also emerged. Base editors allow precise conversion of individual bases without introducing DSBs. For example, Gaudelli et al. (2017) developed adenine base editors (ABEs) that convert A•T to G•C pairs in mammalian cells, with therapeutic potential for correcting point mutations while minimizing off-target effects [12]. Cas13, first described by Gootenberg et al. (2017), cleaves single-stranded RNA without altering DNA, enabling transient modulation of gene expression and inspiring diagnostic tools such as SHERLOCK [13]. Prime editing, introduced by Anzalone et al. (2019), couples a Cas9 nickase with reverse transcriptase and a prime editing gRNA, enabling insertions, deletions, and all possible base substitutions without DSBs or donor templates, thereby, expanding therapeutic possibilities [14]. Most recently, the compact TIGR-Tas system, reported by Faure et al. (2025), uses dual-spacer gRNAs for PAM-independent and nearly unrestricted DNA targeting, representing a potentially transformative addition to the genome-editing toolbox [15].

Nanotechnology has become part of biomedicine through nanomedicine [16,17,18,19,20,21,22,23,24,25,26], a comparatively recent field that applies nanoscale materials and structures to diagnosis, prevention, and treatment, and increasingly intersects with immunoengineering by enabling the design of materials that modulate immune-cell behavior, control antigen or drug delivery, and reshape immune responses in cancer, inflammation, infection, and regenerative medicine [27,28,29,30,31,32,33,34]. For gene therapy, the value of a nanocarrier is not size alone. Composition, charge, morphology, and surface chemistry determine cargo protection, circulation, tissue distribution, cellular entry, and intracellular release [19,35]. Studies in antimicrobial delivery [36,37], including metal-based systems [38], and oral delivery [39] show that nanoparticles can stabilize labile agents and change exposure, but those examples are included here only to establish design principles that also govern nucleic acid and genome-editor delivery.

This review is organized in five linked parts. Section 2 examines how carrier class and editor architecture jointly determine loading, tissue access, cell entry, and cargo release. Section 3 compares gene replacement, programmable nucleases, and RNA-based modalities. Section 4 addresses endosomal escape, intracellular trafficking, and controlled release as shared delivery barriers. Section 5 examines data-guided design, integrated imaging, patient-specific carrier selection, bioprinted test systems, and the manufacturing, safety, and regulatory requirements for clinical use. Section 6 summarizes the main conclusions and translational priorities.

## 2. Nanomaterials Empowering Advanced Genome Editing

Nanomaterials can support genome-editing delivery by protecting nucleic acids and proteins, increasing cellular uptake, and controlling intracellular release [40,41]. Viral and recombinant vectors remain effective, but immunogenicity, insertional risk, and limited cargo capacity can restrict their use for some editors [41,42].

Carrier classes include lipid-based systems such as exosomes and ionizable lipids, DNA-based structures such as nanogels and DNA origami, and polymer carriers such as polyethyleneimine [40,41]; inorganic materials such as gold, silica, and metal–organic materials [43,44]; and carbon-based materials such as nanotubes, graphene, and carbon dots [45,46]. Their behavior is governed by loading chemistry, particle stability, protein adsorption, tissue distribution, and the route by which cargo is released inside the cell.

**Figure 1:**
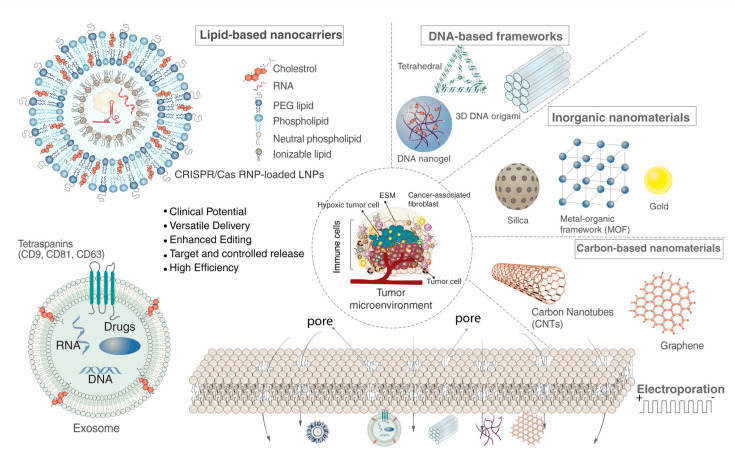
Schematic representation of nanomaterial-based delivery platforms for CRISPR/Cas complexes. Lipid nanocarriers, DNA structures, inorganic materials, carbon-based carriers, exosomes, and physical transfection methods are shown. The tumor microenvironment is presented as a cancer-related example; the carrier classes and entry routes can apply more broadly [24]. The figure does not imply that all platforms have the same tissue distribution, safety record, or clinical maturity.

Figure 1 uses the tumor microenvironment as one disease-specific example of a broader CRISPR/Cas delivery scheme [24]. The carrier classes shown can also be adapted to non-cancer tissues, although biodistribution and safety differ by material and route. Targeting ligands can increase uptake in selected cells [47]. Physical methods such as nanopore electroporation, nanostraws, and magnetofection may bypass some extracellular barriers, but they are generally better suited to local or ex vivo use than to systemic treatment [48].

### 2.1. Next-Generation CRISPR/Cas Variants and Their Delivery Challenges

Cas12 nucleases are used in both molecular diagnostics and genome editing. Target recognition activates sequence-specific DNA cleavage and, in several Cas12 enzymes, collateral cleavage of single-stranded DNA that supports diagnostic detection [49,50]. In genome editing, wild-type Cas12 creates staggered DSBs; nuclease-inactive or fused variants that can regulate transcription or perform base editing without a DSB [50,51].

Cas12 nucleases use a single RuvC-like catalytic domain and, after guide-directed recognition of a target adjacent to the appropriate PAM, generate staggered DNA breaks distal to the PAM [50,51]. Their capacity for guide processing and multiplex editing can simplify some designs [49,50], but nuclease-specific PAM requirements and editor-specific off-target activity must still be considered [51].

Other advanced editing systems introduce unique delivery challenges. Base editors, which fuse deaminases to Cas proteins, allow single-base conversions without DSBs. However, their large fusion protein size complicates viral packaging, making nanomaterial-based carriers essential for efficient delivery [12]. Prime editors, which couple Cas9 nickase with reverse transcriptase, also exceed the packaging limits of AAVs, further highlighting the need for non-viral alternatives such as lipid nanoparticles and polymer nanocarriers [14]. Cas13, which targets RNA, requires carriers that can protect RNA–protein complexes from nuclease degradation, offering opportunities for advanced nanoparticle encapsulation strategies that support transient transcriptome modulation [13]. More recently, the compact TIGR-Tas system introduced PAM-independent DNA targeting. Its dual-spacer gRNA architecture, however, may demand specialized carrier designs to ensure stability and precise co-delivery [15].

The large molecular size of base and prime editors complicates delivery because deaminase or reverse-transcriptase domains increase cargo size. CRISPR activation (CRISPRa) and interference (CRISPRi) create a different delivery problem because nuclease-inactive Cas proteins are fused to transcriptional activators or repressors. No single experimental or computational method predicts all off-target events across these editor classes; the assay must match the mechanism being tested [52]. AAV vectors are also restricted by a packaging capacity of about 4.7 kb, which is insufficient for many multi-domain editors [53,54]. Nanomaterials can instead carry ribonucleoproteins (RNPs), mRNA, plasmids, or donor DNA, although each cargo has different stability, release, and intracellular-routing requirements [41,55].

Delivery performance must be interpreted in relation to cargo form. Cas ribonucleoproteins act quickly and limit the duration of editor exposure, which may reduce prolonged off-target activity, but protein and guide RNA must reach the cytosol together and are vulnerable to degradation [41,55]. Editor mRNA supports transient intracellular protein production and is well suited to LNP loading, whereas plasmid DNA requires nuclear entry and can sustain editor expression for a longer period [56]. A carrier that performs well for one cargo should therefore not be assumed to perform equally well for another.

### 2.2. High-Specificity Delivery Vehicles for Genome Editing

Nanoparticles (NPs) can be engineered to encapsulate multiple molecular components, such as donor DNA and Cas cargo, within a single delivery vehicle, enabling precise spatial and temporal coordination required for HDR [55]. This integrated approach not only minimizes degradation and side effects associated with separate delivery but also increases repair efficiency and enhances the overall quality of genome editing outcomes [57].

Porous nanomaterials, including mesoporous silica and metal–organic frameworks (MOFs), have emerged as promising carriers due to their high loading capacity and controlled release properties [43,44]. Their porous architecture allows encapsulation of therapeutic agents, such as anticancer drugs, while protecting them from premature degradation. At the same time, these carriers facilitate precise, targeted delivery directly into tumor cells, thereby improving therapeutic selectivity [58,59]. By coordinating co-delivery, systemic toxicity is reduced and synergistic therapeutic effects are amplified, especially in tumor microenvironments [60,61].

Delivery of Cas nucleases to non-mammalian systems, such as plants, presents additional challenges. The presence of rigid cell walls serves as a major barrier to the entry of genetic material, complicating the use of CRISPR tools for improving traits such as disease resistance, climate adaptability, herbicide tolerance, and nutritional quality [62,63]. Carbon-based nanomaterials, including carbon nanotubes (CNTs), graphene, and carbon dots, offer unique electrical, thermal, and mechanical properties that enhance cellular uptake, enable controlled release, and reduce cytotoxicity [45]. Unlike traditional methods of plant genetic engineering, CNTs provide a non-invasive and controllable delivery strategy [63].

Due to their nanoscale size, CNTs can traverse plant cell wall size-exclusion limits, enabling delivery of DNA, RNA, and proteins directly into target cells [62,63]. Importantly, their high surface area allows them to protect genetic cargo from enzymatic degradation during delivery, thereby improving the efficiency and stability of genetic modifications [46,64]. Finally, the effectiveness of lipid nanoparticles (LNPs) in supporting CRISPR-based delivery across diverse mammalian tissues has been systematically demonstrated, with several representative studies summarized in Table 1.

Among current nonviral systems, ionizable LNPs have the strongest clinical position because they support reproducible mixing, high nucleic-acid loading, and transient expression [17,70]. Their main limits are liver-biased distribution after systemic dosing, incomplete endosomal release, inflammatory responses to some lipid structures, and uncertain performance after repeated administration [71]. Polymer, DNA, inorganic, carbon, and extracellular-vesicle systems offer broader chemical choices or tissue-specific functions, but most have less standardized production and less human safety evidence.

## 3. Gene Therapy Innovations: From Classic to Next-Generation

### 3.1. Transition from Gene Replacement to Precision Editing

Genome-editing technologies have progressed from early protein-based nucleases to highly programmable RNA-guided systems, enabling precise and efficient genetic modifications. Among these, CRISPR/Cas, zinc finger nucleases (ZFNs), transcription activator-like effector nucleases (TALENs), and meganucleases are the most widely studied platforms for therapeutic applications [72].

CRISPR/Cas systems function through RNA–DNA interactions, using a single-guide RNA (sgRNA) to direct the Cas nuclease to specific genomic loci. In contrast, ZFNs, TALENs, and meganucleases rely on protein–DNA interactions, which require more complex engineering to achieve target specificity [56,72]. For example, CRISPR/Cas employs an sgRNA and Cas9 protein, whereas ZFNs and TALENs require engineered fusion proteins (ZFA–FokI and TALE–FokI, respectively) [53]. Meganucleases are based on restriction enzymes, which are difficult to re-engineer for new target sequences.

The DNA-binding mechanisms also differ among platforms: CRISPR recognizes DNA sequences through sgRNAs, ZFNs employ zinc-finger arrays, TALENs recognize single bases via TALE repeats, and meganucleases require highly specific sequence recognition [53,73]. This distinction makes CRISPR the more versatile and adaptable approach, since sgRNAs can be easily redesigned, whereas protein-based platforms require extensive re-engineering for each new target site [49].

From a delivery perspective, CRISPR/Cas has an open reading frame (ORF) size of approximately 4.2 kb (including sgRNA), compared with 2.1 kb for ZFNs, 2.2 kb for TALENs, and 1.1–4 kb for meganucleases [54]. The smaller size of ZFNs and meganucleases makes them easier to deliver using viral vectors [74]. By contrast, CRISPR and TALENs are often delivered using AAVs or lentiviruses, while ZFNs and meganucleases are primarily delivered via AAV systems.

Another key distinction is in target-sequence requirements. CRISPR recognizes a 22 bp sequence followed by a PAM, whereas ZFNs and TALENs require longer recognition sequences (18–40 bp), making their design and validation more complex [75].

**Figure 2:**
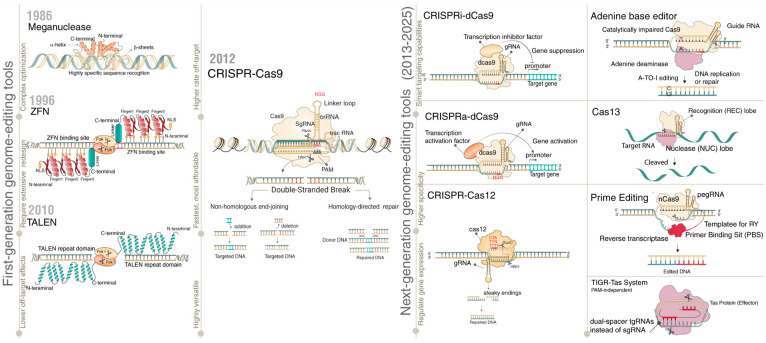
Evolution/Timeline of genome editing tools. Genome editing progressed from protein–DNA systems—Meganucleases (1986), ZFNs (1996), and TALENs (2010) to the RNA-guided CRISPR-Cas9 (2012), which streamlined targeting and increased versatility. From 2013 through 2025, CRISPRi/a, Cas12, base editors, Cas13, prime editing, and TIGR-Tas came on the scene, each providing increased precision and wider applications. Collectively, they drastically reformed genome engineering, increasing programmability, efficacy, and therapeutic potential and transitioning from complicated protein-based designs towards simpler and more versatile RNA-guided technologies continuing to define biomedical research.

The evolution of genome-editing technologies from early protein–DNA systems to RNA-guided CRISPR and next-generation modalities is illustrated in Figure 2.

Another major advantage of CRISPR, particularly when integrated with nanomaterials, is its compatibility with large-scale library construction, enabling high-throughput genome editing applications [46]. In contrast, ZFNs, TALENs, and meganucleases face significant barriers in generating diverse libraries due to the complexity of engineering their protein components [41].

CRISPR also demonstrates relative insensitivity to DNA methylation, whereas ZFNs, TALENs, and meganucleases are strongly affected by methylation status, potentially limiting their effectiveness in epigenetically modified genomic regions [7]. Further, CRISPR offers superior speed and cost efficiency, typically requiring only 1–3 days for design and implementation [49,50]. By comparison, ZFNs and TALENs require 5–15 days and are more expensive due to the complexity of protein engineering, while meganucleases demand extensive optimization, making them less cost-effective overall [76].

Off-target activity cannot be assigned to one editing platform in general. It depends on nuclease architecture, target sequence, dose, duration of expression, chromatin context, and the detection method [51,52]. Guide-RNA mismatch tolerance can cause unintended CRISPR cleavage, but high-specificity Cas variants, guide design, and transient RNP delivery can reduce exposure [7,77]. ZFNs, TALENs, and meganucleases can also cleave related sequences, and their dimeric or protein-engineered designs do not guarantee lower toxicity [78]. Comparisons are therefore informative only when platforms are tested at the same locus, dose, cell type, and assay sensitivity.

Wild-type Cas9, ZFNs, TALENs, and meganucleases generally create DSBs [73,74]. Cas9 nickases create single-strand breaks, while base and prime editors use modified Cas proteins to avoid or reduce DSB formation [12,14]. Repair outcome depends on break structure, cell cycle, donor availability, and local sequence context rather than on platform name alone. NHEJ often dominates after a DSB, whereas precise HDR usually requires donor DNA and is less efficient in nondividing cells [72,73]. Delivery affects both efficacy and safety because cargo form and dose determine how rapidly editing begins and how long nuclease activity persists [7]. Beyond these programmable nuclease systems, polymer- and DNA-based carriers provide distinct options for RNP and donor delivery, as compared in Table 2.

Polymer carriers allow close control of charge density, degradability, and ligand display, and they can carry RNPs or donor DNA that are difficult to package in viral vectors [42,84]. DNA nanostructures can encode aptamers, responsive sequences, and several nucleic-acid functions in one assembly, but they face nuclease stability and multi-step production limits [80,81]. For synthetic polymers, molecular-weight distributions, residual monomers, formulation heterogeneity, and cationic toxicity may vary between batches. Clinical use therefore depends on degradable chemistry, reproducible synthesis, and direct comparison at matched dose and cargo.

### 3.2. Emerging Modalities: RNA, mRNA, and Beyond

Messenger RNA (mRNA) represents the fundamental code for protein synthesis, and its integration with nanomaterials is transforming personalized medicine. Nanotechnology can enhance mRNA stability, enable precise delivery, and improve translational efficiency, thereby overcoming critical therapeutic challenges [88]. Nanoparticle-based carriers, LNPs, have markedly improved mRNA delivery by protecting cargo from enzymatic degradation and enhance targeted cellular entry. This progress also enables the engineering of designer fusion proteins that combine multiple functional domains to regulate cell signaling and metabolism, including strategies for targeted protein degradation via ubiquitination [89].

Efficient nucleic acid delivery requires not only entry into target cells but also localization to the correct subcellular organelle, which remains a major therapeutic challenge [89]. NPs must be directed to specific cells, a process that can be enhanced using antibody functionalization to boost selective uptake [25,90,91]. Following uptake, NPs must also escape the endosomal pathway to release cargo into the cytoplasm [92]. The biological activity of nucleic acids depends heavily on their location. Cytoplasmic-acting nucleic acids such as small interfering RNA (siRNA), antisense oligonucleotides (ASOs), and mRNA operate through distinct mechanisms; siRNA facilitates RNA-induced silencing complex (RISC)-mediated degradation of target mRNA, ASOs either recruit RNase-H for degradation or interfere with translation, and mRNA is directly translated into a therapeutic protein [93]. In contrast, for stable expression, DNA transgenes require nuclear entry, incorporation into the genome, transcription into mRNA, and subsequent translation in the cytoplasm [94]. Nucleic acids can also be targeted to mitochondria, where RNA is locally translated into mitochondrial proteins or DNA is used to replace mutated mitochondrial genomes [95]. The rapid development of mRNA vaccines during the COVID-19 pandemic demonstrated the clinical potential of nanoparticle-based mRNA delivery for infectious disease prevention and immunotherapy [20,93].

siRNAs and microRNAs (miRNAs) are short, non-coding RNAs that play critical roles in post-transcriptional gene silencing through RNA interference (RNAi). Both regulate gene expression by inducing mRNA degradation and/or blocking translation of mRNA in the cytoplasm [96]. siRNAs are typically 21–23 nucleotides in length and originate from double-stranded RNA precursors [97]. Once introduced into cells, these precursors are cleaved by Dicer into siRNA fragments [98]. A single strand of the siRNA duplex is then incorporated into the RISC [99], where it directs binding to complementary mRNA sequences, resulting in cleavage and degradation of the target transcript [100].

miRNAs, produced endogenously, follow a distinct maturation pathway. They are initially transcribed as primary miRNAs (pri-miRNAs), processed into precursor miRNAs (pre-miRNAs), and exported into the cytoplasm, where Dicer processes them into mature miRNAs [98]. Like siRNAs, miRNAs are loaded into the RISC; however, instead of inducing direct cleavage, miRNAs typically bind to partially complementary sequences within the 3′ untranslated region (UTR) of target mRNAs, leading to translational repression [93].

The RISC plays a central role in RNAi by guiding sequence-specific targeting of mRNAs [93]. Within the RISC, the guide strand is retained while the passenger strand is degraded [101]. The core protein, Argonaute (AGO), mediates target recognition and cleavage [102]. In siRNA pathways, AGO binding to fully complementary target sequences induces mRNA cleavage and rapid degradation, silencing gene expression [103]. In contrast, the miRNA-loaded RISC generally results in translational repression, with target mRNAs stored in processing bodies (P-bodies) for degradation or maintained in a repressed state [104,105]. The efficiency of RISC-mediated silencing depends on several factors, including guide–target complementarity, associated cofactors, and cellular conditions [106].

ASOs are short, synthetic, single-stranded DNA molecules designed to bind complementary RNA sequences through Watson–Crick base pairing, thereby inhibiting expression of specific target genes [107]. ASOs exert their effects through two primary mechanisms: steric block ASOs, which interfere with ribosome binding, RNA splicing, or stability, and RNase-H–competent ASOs, which recruit RNase-H to degrade the RNA strand of RNA–DNA heteroduplexes [108]. Steric block ASOs bind mature mRNAs in the cytoplasm or pre-mRNAs in the nucleus, while RNase-H–competent ASOs act in both compartments to degrade target mRNA [109]. This targeted degradation reduces protein expression, making ASOs a powerful therapeutic tool for gene silencing [110]. Lastly, ASOs have gained significant interest as therapeutic candidates for genetic disorders and viral infections [109], and for cancer [111,112], with the advantage of reduced off-target effects compared to other gene-editing modalities.

## 4. Overcoming Key Barriers in Advanced Gene Therapy

### 4.1. Endosomal Escape and Intracellular Trafficking

NPs typically enter target cells through endocytosis, where one of the most significant challenges is endosomal entrapment. Endocytosis-mediated uptake often results in sequestration of NPs within endosomes and subsequent degradation in lysosomes, which severely limits delivery efficiency [3,41]. But several mechanisms have been explored to improve endosomal escape. These include the use of cationic polymers, zwitterionic lipids, bio-reducible disulfide linkages, and other chemical modifications that promote membrane destabilization [41]. NPs can also escape via the proton sponge effect or by inducing pore formation in the endosomal membrane, both of which facilitate cytoplasmic release [48].

**Figure 3:**
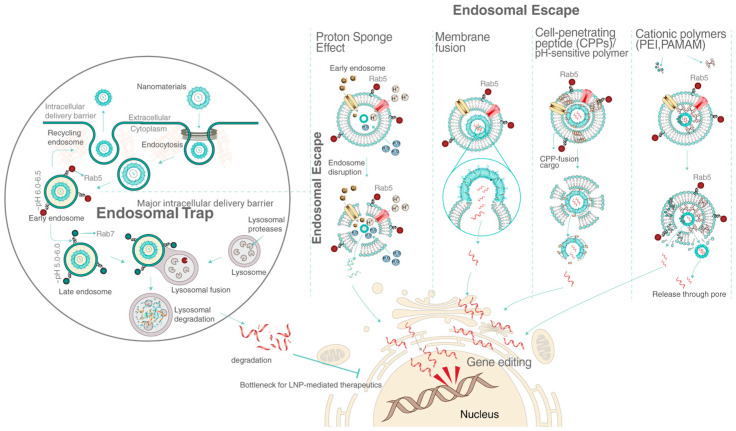
Mechanisms of endosomal entrapment and escape of nanomaterial-based CRISPR/Cas delivery. Cellular engulfment of nanocarriers by endocytosis traps them within early and late endosomes, which usually results in lysosomal digestion. Mechanisms of escape are the proton sponge effect (rupturing of endosomes), membrane fusion, cell-penetrating peptides (CPPs) or pH-sensitive polymers, and cationic polymers (such as PEI, PAMAM). Successful endosomal escape is vital for effective delivery of gene-editing cargo into the nucleus.

Physical transfection techniques represent another route of entry, in which temporary membrane destabilization reduces pore size and enhances cytosolic delivery, although this approach is less commonly applied than chemical strategies [41,49]. Importantly, once internalized, nanocarriers must escape from endosomal and lysosomal pathways to maintain the therapeutic activity of their cargo. Thus, endosomal entrapment is recognized as one of the primary barriers to efficient nanomaterial-based delivery [113]. The mechanisms of endosomal sequestration and strategies for cytoplasmic escape are illustrated in Figure 3. Current delivery methods remain highly inefficient, with as little as 1% of delivered cargo successfully reaching its intracellular target. Strategies under investigation include fusion with endosomal membranes, incorporation of destabilizing polymers, and induction of the proton sponge effect, though recent evidence suggests that the proton sponge mechanism may play a less dominant role than previously believed [113,114].

Membrane fusion is one important strategy for endosomal escape. Specific lipids can merge with endosomal membranes, enabling lipid-encapsulated NPs to release their cargo into target cells [115]. NP formulations can also be engineered to directly disrupt endosomal membranes, destabilizing their structure and enhancing cytosolic delivery [116]. In addition, a variety of pH-sensitive polymers and cell-penetrating peptides have been investigated to improve intracellular trafficking of therapeutic payloads [117]. Poly(amidoamine) (PAMAM) dendrimers, for example, induce osmotic swelling of endosomes, a phenomenon often described as the proton sponge effect, which has been linked to enhanced cytosolic release. However, recent studies suggest that membrane fusion and destabilization are the dominant mechanisms underlying effective endosomal escape [113].

A major barrier to optimizing these strategies is the lack of reliable methods measuring endosomal escape. Current evaluation techniques rely heavily on endpoint assays, such as assessing downstream gene expression or knockdown, which do not provide direct information about the escape process [117]. As a result, drug delivery outcomes are often difficult to interpret, and inefficient endosomal release remains a bottleneck for nanomaterial-based systems [118,119].

To address this, several ex vivo and in vitro assays have been developed. Dye-loaded liposomes are commonly used to mimic endosomal membranes and measure disruption by nanomaterials [119]. Similarly, red blood cell lysis assays are employed to test membrane-disruptive potential of carriers for genome engineering applications [120,121]. Red blood cell membranes, being more complex than synthetic liposomes due to their protein and carbohydrate components, may serve as more realistic biological models [121,122]. It is also important to note that endo-lysosomal membranes differ significantly from plasma membranes in their lipid and protein composition, which influences nanoparticle escape efficiency [123]. For genome-editing applications such as CRISPR-Cas9, efficient cytoplasmic and nuclear delivery is essential, making endosomal escape a critical determinant of editing success [124].

Fluorescence-based localization assays are frequently used to evaluate nanocarrier success, helping researchers refine designs to minimize degradation and improve delivery outcomes [125,126]. However, standard in vitro fluorescence localization assays often face challenges in accurately identifying endosomal escape, since fixation can disrupt membranes and misrepresent escape events compared to live-cell imaging [127,128]. Escape is typically inefficient, and weak signals are often masked by strong background fluorescence from the endo-lysosomal compartment [121]. Further, absence of colocalization with endosomal markers does not necessarily confirm cytosolic release, since cargo may be sequestered in alternative vesicles [113,127].

Calcein, a fluorescent dye, is widely used as an escape indicator with punctate fluorescence denoting endosomal entrapment, while diffuse cytoplasmic fluorescence reflects successful release [127]. Other methods, including fluorescence spectroscopy and split-GFP complementation assays, have provided additional insights but still suffer from limited sensitivity and accuracy [129]. More recently, the SLEEQ (Sensitive Light-based Evaluation of Endosomal Escape Quantification) assay enabled more precise quantification of NP escape efficiency, advancing the development of optimized nanomaterials for genome engineering applications [130,131].

Total cellular uptake is a poor substitute for cytosolic delivery [113,127]. A carrier may accumulate in cells yet release little active cargo, whereas a formulation with lower uptake can produce more editing if release occurs before lysosomal degradation [130,131]. Direct escape assays should therefore be paired with cargo-specific measurements such as cytosolic RNP availability, editor-expression kinetics, and on-target editing at matched dose.

### 4.2. Precision Dosing and Controlled Release

Enveloped viruses possess an outer lipid bilayer that merges with host cell plasma membrane, allowing them to release their nuclei acid into the cytoplasm, where replication and infection begins [132,133]. This natural mechanism has inspired strategies for nucleic acid delivery. For example, the fusogenic lipid DOPE (1,2-dioleoyl-sn-glycero-3-phosphoethanolamine) promotes membrane fusion through lipid inversion, a process confirmed using synchrotron small-angle X-ray scattering and NMR spectroscopy [134]. Molecular dynamics simulations have further revealed how unilamellar vesicles, small, single-layered lipid structures, merge with cellular membranes to facilitate drug delivery. Charged lipids that form NPs, such as LNPs and liposomes, can undergo lipid inversion, which enables controlled intracellular release of therapeutic molecules [23,134,135].

The success of SARS-CoV-2 vaccines highlighted the clinical relevance of LNPs. Both the Pfizer/BioNTech (BNT162b2) and Moderna (mRNA-1273) vaccines relied on proprietary lipid formulations designed to promote cytosolic delivery, stability, and efficient immune activation [136,137]. Cationic lipids interact with anionic phospholipids in endosomal membranes, driving lipid inversion and the formation of a hexagonal (HII) phase, which destabilizes membranes and promotes cargo release [138,139].

Ionizable lipids are now widely used in LNP construction because they become protonated at endosomal pH, mimicking the fusogenic behavior of cationic lipids while reducing systemic toxicity [71]. However, despite protecting nucleic acids from degradation, LNPs still exhibit inefficient endosomal escape, with studies showing siRNA and mRNA delivery efficiencies below 5% and 1%, respectively, and largely due to poor escape and rapid recycling [71].

To overcome these limitations, multiple strategies have been investigated to enhance lipid-based delivery such as structural modifications to the hydrophilic head group, hydrophobic tails, or linker regions, all shown to alter membrane destabilization properties [140]. Researchers have also developed combinatorial lipid libraries to systematically evaluate how structural variations in ionizable lipids influence endosomal escape [141,142]. Representative examples of these systems are summarized in Table 3.

Clinical maturity is not uniform across these systems. Ionizable LNPs should receive the highest translational priority for systemic RNA or editor-mRNA delivery because human production and safety experience already exist [17,70]. Cationic and PEGylated liposomes remain useful for local administration or prolonged circulation [146,148], but permanent positive charge and anti-PEG immunity can limit repeat dosing. Stimuli-responsive and hybrid systems may improve tissue control or intracellular release [152,155], while polymer and polypeptide carriers can provide degradable or receptor-directed delivery [84,162]. Most of these added-complexity systems remain preclinical because each component increases characterization, scale-up, and regulatory requirements [157,163].

## 5. Future Directions and Translational Priorities in Nanomaterial-Enabled Genome Editing

Future research should connect carrier selection, biological validation, and clinical production rather than treat them as separate topics [41,48]. Data-guided screening can reduce the number of formulations that require experimental testing, but selected candidates still need confirmation in disease-relevant cells, animal models, and human-derived tissues. Imaging can then establish tissue distribution and persistence [164], while patient data may guide carrier and dose selection. Bioprinted tissues can support this evaluation before clinical studies. Progress to clinical use depends on defined composition and reproducible production [165,166], as well as safety after repeat dosing and regulatory evidence for both the carrier and the editing cargo [164,167].

### 5.1. AI-Assisted Nanocarrier Design

Data-guided design is most useful when it links material composition to a defined biological measure. Yamankurt et al. tested nearly 1000 spherical nucleic-acid formulations across 11 design variables and used machine learning to identify structure-activity relations associated with immune activation [168]. Kumar et al. combined parallel polymer synthesis with machine learning to relate polymer chemistry to CRISPR RNP uptake, editing, and cytotoxicity, leading to a polymer carrier with improved RNP delivery [169]. These studies show that computational models can reduce experimental search while still requiring direct biological confirmation.

More recent studies have focused on LNPs. Wang et al. trained models to predict mRNA-LNP performance from lipid structure and formulation data [170]. Li et al. coupled a 584-lipid library with machine learning and virtual screening to identify ionizable lipids for mRNA delivery [171]. The AGILE method combined deep learning with combinatorial chemistry and found that lipid features associated with delivery differed between HeLa cells and macrophages [172]. A separate study screened nearly 20 million virtual ionizable lipids, then synthesized selected candidates that matched or exceeded commonly used control lipids in mice [173].

Machine learning has also been used to prescreen millions of lipidic carriers for mRNA delivery and to derive cell-type-specific composition rules from 1080 plasmid-DNA LNP formulations tested across six cell types [174,175]. These results are concrete, but current models remain limited by small datasets, inconsistent reporting, assay-specific labels, and weak transfer from cell lines to human tissue. Models should therefore be evaluated on external data, and studies should report inactive formulations as well as successful ones. At present, these methods support candidate selection; they do not replace pharmacology, toxicology, or production studies.

### 5.2. Integrated Delivery, Monitoring, and Patient Selection

Imaging and patient selection are most useful after a carrier has shown adequate delivery. Imaging labels or reporter cargo can establish where particles accumulate, how long they persist, and whether editor activity remains confined to the intended tissue [21,176]. These measurements can inform dose, route, and eligibility criteria, but adding an imaging agent changes particle composition and can complicate production and regulatory review.

Patient-specific use should begin with clinical variables that can affect delivery, such as target-cell abundance, receptor expression, liver function, inflammatory status, and pre-existing antibodies to carrier components [17,70]. A separate formulation for every patient is unlikely to be practical. A more realistic strategy is to select among a small set of well-characterized carriers and dosing routes, supported by imaging and tissue-specific biomarkers [165,166].

### 5.3. Bioprinted Models for Preclinical Evaluation

Bioprinted tissues are best used as controlled preclinical test systems rather than as an immediate route for treating patients. They can place defined human cell types, extracellular matrix, and spatial structure in the same model, allowing comparison of nanoparticle penetration, cell-type selectivity, editing, and local toxicity under conditions that are more informative than two-dimensional culture [177].

The present limits are substantial. Printed tissues often lack mature vasculature, immune components, and long-term physiological function, and nanoparticle behavior can change with matrix composition and printing chemistry. Their value will depend on comparison with animal and human tissue data and on models that measure both successful editing and unintended effects [177,178].

### 5.4. Safety, Manufacturing, and Regulatory Requirements

For clinical use, ionizable LNPs currently have the strongest case for systemic RNA and editor-mRNA delivery because they have established production methods and human exposure data [17,41]. Their limits include liver-dominant distribution, incomplete endosomal release, innate immune activation, anti-PEG responses, and uncertainty after repeated dosing [71]. Polymer carriers may be better suited to local delivery, ex vivo cell engineering, or cargo combinations that exceed LNP loading constraints, but composition and molecular-weight variability must be controlled [179]. Inorganic and hybrid particles can support imaging or externally triggered release, yet persistence, metal-related toxicity, and multi-component production make clinical assessment more difficult [26,180].

Regulatory assessment must address both the nanocarrier and the editor. Required evidence includes identity and purity of each component, particle-size and charge distributions, encapsulation, release, potency, sterility, stability, biodistribution, shedding, immunotoxicity, genotoxicity, and off-target editing [165,166]. Changes in lipid source, polymer molecular weight, mixing conditions, or scale can change biological performance even when average particle size appears similar [164,165]. For this reason, production controls and potency assays should be linked to the mechanism of delivery, and long-term follow-up should reflect the persistence of both carrier and editing effect [167].

## 6. Conclusions

Nanomaterial-based delivery is most advanced where the cargo is transient and the carrier can be produced reproducibly. Ionizable LNPs currently have the greatest translational and therapeutic potential for mRNA, siRNA, and editor-mRNA delivery because they combine high loading, scalable mixing, and human clinical experience. Their strongest evidence is in liver-directed delivery; reliable delivery to the brain, lung, muscle, solid tumors, and hematopoietic tissues remains less consistent.

Polymer carriers are a strong second group for local administration, ex vivo cell engineering, and co-delivery of RNPs with donor DNA. They offer chemical control over degradation and release, but batch variation, residual reagents, and cationic toxicity still limit clinical use. DNA nanostructures, extracellular vesicles, inorganic particles, carbon materials, and hybrid systems provide useful functions in selected settings, yet most remain earlier in development and require clearer evidence on clearance, repeated dosing, and production.

Across all carrier classes, the main unresolved problems are extrahepatic tissue targeting, efficient endosomal release, dose control, immune responses, long-term safety, and direct comparison at matched cargo and dose. Clinical progress also requires stable production at scale, validated potency assays, and regulatory plans that assess the carrier and editor as one product.

The most productive direction is therefore not a single universal carrier, but a limited set of well-characterized systems matched to editor size, cargo form, target tissue, and treatment route. This pairing is especially important for base editors, prime editors, Cas13 systems, and compact RNA-guided nucleases, whose delivery needs differ from those of conventional Cas9.

## Figures and Tables

**Table 1:** Comparison of Lipid Nanoparticle-Based CRISPR Delivery Systems.

LNP Application	Outcome or Efficiency	Major Advantage	Major Limitation	Clinical Status	References
Cas9 RNP delivery to mouse corneal tissue	Efficient local editing; exact percentage not reported	Local dosing limits systemic exposure	Local ocular administration does not establish systemic delivery or performance in other tissues	Preclinical	[65]
Microfluidic LNP delivery of CRISPR RNP	Base substitution up to 23%; gene disruption up to 97%	Reproducible mixing and high editing in tested systems	RNP stability and large-scale comparability require further study	Preclinical	[66]
Ultrasound-controlled LNP with sonodynamic therapy	Improved antitumor activity under ultrasound	External control of cargo release	Requires accessible tissue and a multi-component product	Preclinical	[67]
DOTAP and organ-selective LNP formulations	>80% editing in selected tissues in reported models	Composition can shift tissue distribution	Permanent cationic charge and formulation-dependent tropism may limit repeat dosing	Preclinical	[68,69]
LNP-mediated in vivo editing for transthyretin amyloidosis	Human in vivo editing with marked TTR reduction	Direct clinical evidence for systemic LNP editor delivery	Predominantly liver-directed; long-term and repeat-dose questions remain	Clinical	[70]

**Table 2:** Comparison of Polymer- and DNA-Based CRISPR/Cas9 Delivery Systems.

System	Material and Targeting Design	Cargo, Target, and Reported Result	Major Advantage	Major Limitation	References
Cas9-P nanocarrier	Branched PEI with phosphorothioate-modified DNA	Cas9 RNP targeting PD-L1; tumor suppression in melanoma	Strong complex formation and cellular uptake	Cationic toxicity and polymer heterogeneity	[79]
DNA nanoflower	DNA structure with MUC1 aptamer and miR-21-responsive sequences	Cell-selective release and editing linked to miR-21	Targeting and response elements can be encoded in DNA	Nuclease stability and multi-step production	[80]
Ultra-long ssDNA particle	Rolling-circle DNA with DNAzyme motifs and Mn2+ compaction	Co-delivery of CRISPR/Cas9 and DNAzyme	Carries several nucleic-acid functions in one particle	Purity, scale, and sequence-dependent assembly	[81]
FA-PEG oligoamino amide	Folate ligand and PEG for receptor-directed delivery	PD-L1 and PVR disruption in CT26 tumor models	Dual targeting and checkpoint editing	Receptor heterogeneity and anti-PEG responses	[82]
mPEG-PC7A particle	pH-sensitive amphiphilic polymer	Editing through NHEJ or HDR in tested models	Charge and hydrophobicity can be adjusted	Performance is formulation-dependent; limited human data	[83]
Biodegradable nanocapsule	Mixed-charge polymer with imidazole and reduction-sensitive crosslinks	Robust in vitro and in vivo RNP editing with low cytotoxicity	Degradable chemistry and intracellular release	More components increase production and characterization demands	[84]
Angiopep-2 polymer particle	Angiopep-2 with guanidinium and fluorine groups	PLK1 editing in glioblastoma models; 32% knockout/narrative introducing brain-tumor delivery	Brain-targeting strategy with RNP stabilization	Blood–brain barrier models may not predict human delivery	[22,85]
Phenylboronic dendrimer	Boronic-acid-rich dendrimer with hyaluronic acid coating	APC and KRAS editing in colorectal cancer models	High protein loading and tumor-associated targeting	Clearance, cationic toxicity, and batch control require study	[86,87]

**Table 3:** Comparative Features of Synthetic Delivery Systems for CRISPR Components.

Type	Mechanism or Key Feature	Major Advantage	Major Limitation	Representative Use	Clinical Maturity	References
Cationic liposomes	Electrostatic cargo binding and membrane interaction	High loading and cellular entry	Permanent charge can increase serum interactions and toxicity	Cas9/sgRNA delivery and aptamer-directed cancer targeting	CRISPR use remains preclinical	[143,144,145]
PEGylated liposomes	PEG reduces rapid clearance; ligands can direct uptake	Longer circulation and surface modification	Anti-PEG antibodies and reduced cell entry after dense PEG coating	Brain and tumor-directed plasmid delivery	Established liposome production; CRISPR preclinical	[146,147,148]
Fusogenic liposomes	Lipid mixing with cellular or endosomal membranes	Direct intracellular release	Fusion depends strongly on lipid composition and biological membrane state	Liposome-exosome and membrane-fusogenic systems	Preclinical	[149,150,151]
Stimuli-responsive liposomes	Release triggered by pH, light, or reactive oxygen species	Spatial or temporal control	Requires an external trigger or a reliable disease-specific signal	Light- and pH-controlled Cas9 delivery	Preclinical	[152,153,154]
Hybrid liposomes	Lipids combined with polymers, silica, or gold	Can combine loading, targeting, and triggered release	Multi-component production and safety assessment are more difficult	Photothermal and liver-directed editing systems	Preclinical	[154,155,156,157]
Ionizable LNPs	Charge develops at acidic pH, supporting loading and endosomal membrane interaction	Scalable mixing and strongest human experience for nucleic acids	Liver bias, incomplete escape, inflammatory effects, and repeat-dose concerns	Cas9 mRNA/sgRNA and in vivo liver editing	Highest translational maturity	[158,159,160,161]
Polymer nanoparticles	Reduction-sensitive or pH-sensitive intracellular release	Broad chemical control and RNP compatibility	Batch variation, residual reagents, and cationic toxicity	Biodegradable RNP nanocapsules	Preclinical	[84]
Polypeptide carriers	Protease-sensitive or reduction-sensitive peptide release	Biodegradable and suitable for ligand display	Proteolysis, limited stability, and complex scale-up	Tumor- and liver-directed RNP delivery	Preclinical	[162,163]

## Data Availability

No new data were created or analyzed in this study. Data sharing is not applicable to this article.
